# A Case Report of Bacteremia Due to a Symptomatic and Rare Lactobacillus Rhamnosus Infected Splenic Hematoma and the Ultimate Treatment Model

**DOI:** 10.7759/cureus.36128

**Published:** 2023-03-14

**Authors:** Margaret Kell, Zachary Carter Lee, Michelle Hernandez, Marsha Crader, John Norwood

**Affiliations:** 1 Department of Internal Medicine Residency Program, St. Bernards Regional Medical Center, Jonesboro, USA; 2 Pharmacology, University of Arkansas for Medical Sciences College of Pharmacy, Little Rock, USA; 3 Department of Pharmacology, St. Bernards Regional Medical Center, Jonesboro, USA; 4 Department of Infectious Diseases, St. Bernards Regional Medical Center, Jonesboro, USA

**Keywords:** antibiotic treatment, bacteremia, lactobacillus rhamnosus, gut flora, splenic hematoma

## Abstract

We present the case of a 76-year-old male who presented to our hospital with a rare infection of *Lactobacillus rhamnosus. *The patient had a suspected urinary tract infection (UTI) secondary to a chronic indwelling catheter; however, when symptoms did not improve on standard therapy, blood cultures revealed the growth of *L. rhamnosus*. The patient was found via imaging to have a concurrent infectious splenic hematoma, and aspiration confirmed the presence of *L. rhamnosus.* The patient resided in an area nursing home and was a poor historian; however, it is conceivable that this infection was acquired via diet or from normal gut flora as the patient did not present on probiotic supplementation. In this case report, we present both pharmaceutical and interventional treatment strategies as well as a timeline of treatment for this rarely-seen infection.

## Introduction

*Lactobacillus* is a genus of 261 unique species that are characterized as gram-positive, anaerobic, and aerotolerant bacteria. These bacteria are generally considered beneficial and are found in humans as part of the protective normal flora within the oropharynx, gastrointestinal (GI) tract, and female genitourinary tract. In addition, Lactobacilli are utilized in the fermentation of foods to enhance their nutritional value [1,2].* Lactobacillus rhamnosus* specifically has been identified in vegetables, cereals, sewage, and humans. Within humans, it is part of the normal flora and is seen in the mouth, GI tract, and vagina [2]. Infection with *L. rhamnosus* is rare, with the literature citing only 13 combined cases of bacteremia, endocarditis, liver abscesses, and other infections from the years 2019 through 2021. Cases of Lactobacilli causing abscesses are documented; however, prior treatment with probiotics is often noted [1,3]. 

We present a patient who was treated for an extended-spectrum beta-lactamase positive (ESBL+) *E. coli* UTI with limited improvement upon antibiotic adjustments following susceptibility data. Further investigation identified a splenic hematoma infected by *L. rhamnosus* as the causative source of bacteremia in the patient. The patient, though a poor historian, denied taking any probiotic supplementation, and therefore it is believed that this patient likely acquired this infection secondary to dietary intake or as opportunistic normal flora in the patient’s GI tract [4]. As infection with this bacteria is rare, we present this case as a means to delineate appropriate treatment given current literature and clinical understandings of antibiotic susceptibility in this patient. 

## Case presentation

We discuss a patient who is a 76-year-old nursing home resident with a past medical history of coronary artery disease, intracerebral hemorrhage, peripheral vascular disease, hypertension, hyperlipidemia, chronic obstructive pulmonary disease, type 2 diabetes mellitus, atrial fibrillation, congestive heart failure, neurogenic bladder, pacemaker placement, and dementia who came to our facility via emergency medical services due to nausea, vomiting, and fever. He was found in the Emergency Department to have suprapubic tenderness and a chronic indwelling urinary catheter. On admission, the patient reports nausea and vomiting as well as decreased appetite and intake. 

Vital signs upon admission were significant: a temperature of 103.2 ^o^F, blood pressure of 138/98 mmHg, a respiratory rate of 21, O2 saturation of 95%, and a pulse of 80 bpm. Initial laboratory analysis was significant for white blood cells of 22.6, hemoglobin 11.5, hematocrit 36.4, neutrophils of 90%, fibrinogen of >700 (range 279-571 mg/dL), blood urea nitrogen of 26, creatinine of 1.6, phosphorous of 2.2 (range 2.5-4.5 mg/dL), alk phos of 137, and troponins of 0.041, 0.057, and 0.061. Lactic acid 1.7. A urinalysis was found to have positive nitrites, moderate leukocyte esterase, >50 WBCs, 4+ bacteria, and >1000 glucose. 

The patient was started on empiric antimicrobial coverage with IV piperacillin/tazobactam 3.375 grams, and there was a clinical improvement after initiation. Preliminary blood cultures were initially negative. Leukocytosis improved initially, with a decrease in WBCs to 10.6 (ref. 4.8-10.8 10^3^/uL). Urine culture revealed the growth of ESBL (+) *Escherichia coli*. Once susceptibility results for the ESBL (+)* E. coli *were received, the patient’s regimen was changed to IV meropenem 1 gram; however, continued clinical improvement was not observed in the patient, and there was noted to be a rebound of leukocytosis. Initial and repeat peripheral blood cultures ultimately revealed the growth of* Lactobacillus rhamnosus. *

**Figure 1:**
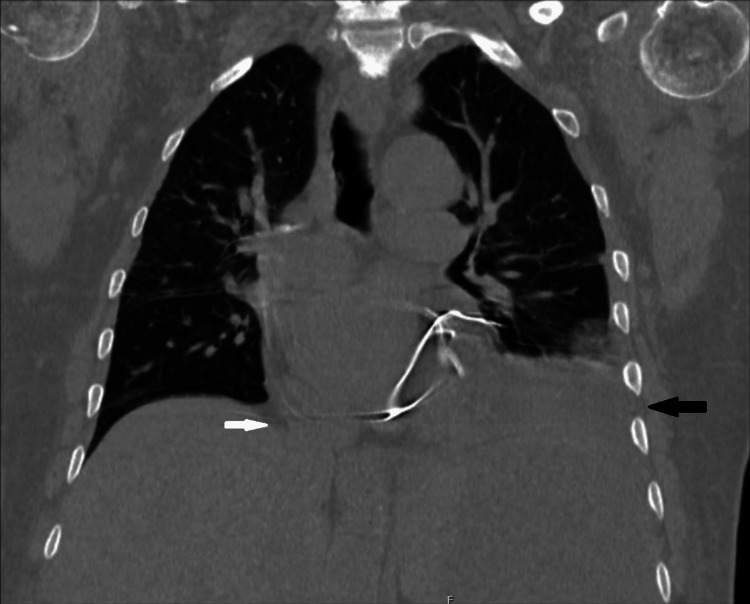
CT chest without contrast reveals pleural effusions and a large splenic mass CT chest without contrast reveals bilateral pleural effusions as well as a large splenic mass not seen on previous imaging at our facility.

**Figure 2:**
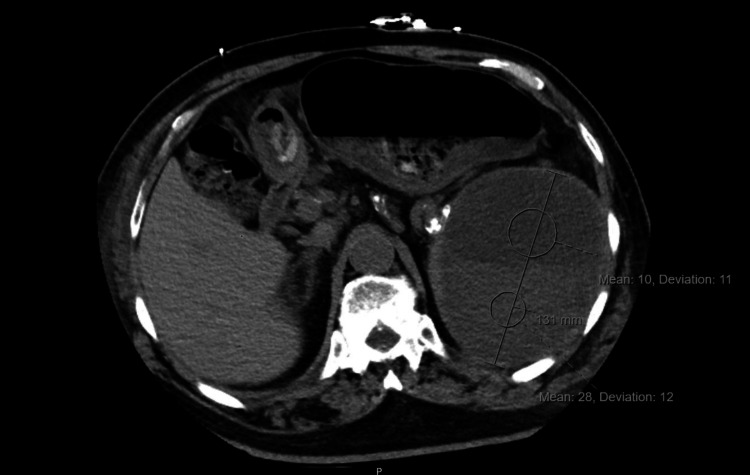
CT abdomen and pelvis reveal a new splenic fluid level

Imaging studies were obtained as the patient’s bacteremia and leukocytosis persisted. A CT scan of the chest revealed a splenic mass and bilateral pleural effusions (Figure 1). A CT of the abdomen and pelvis revealed a 13.1 cm subcapsular fluid collection of the spleen with identifiable fluid levels that were new from prior imaging available at our facility two months prior (Figure 2). Interventional radiology was then consulted for fluid drainage. Bloody fluid was appreciated upon drainage and was sent for culture with* L. rhamnosus *identified.

Given the scarcity of literature regarding the treatment of lactobacilli-related bacteremia, a review of the literature was conducted to assess a treatment plan for the patient. In addition, cultures of both blood and aspirated perisplenic fluid were sent to a reference laboratory for susceptibility testing. Antibiotic susceptibilities were tested and included the following, found in Table 1, for blood and splenic drainage:

**Table 1:** Reported antibiotic susceptibilities of blood cultures and splenic fluid Antibiotic sensitivities showed a variety of antibiotics to which *L. rhamnosus* should be sensitive. Given the patients clinical response and class sensitivity, piperacillin/tazobacam was provided as an outpatient along with percutaneous perisplenic drainage. Treatment recommendations included antibiotics and drainage for 4-8 weeks, depending on the clearance of infection as seen on CT imaging. At the 4-week interval checkpoint, infection reduction was seen on CT imaging, with concomitant therapy reducing the perisplenic fluid collection from 13.1 cm during hospitalization to 4.9 cm. Based on these sensitivities and imaging findings, a full 8-week treatment course was provided to the patient with piperacillin/tazobacam and drainage. The above reference laboratory MIC breakpoint interpretations were based on Food and Drug Administration or Clinical Laboratory Standards Institute recommendations.

Antibiotic MIC	Sensitivity	Source
Ampicillin MIC=2	Sensitive	Blood, Splenic Fluid
Clindamycin MIC	Sensitive	Blood, Splenic Fluid
Daptomycin MIC	Sensitive	Blood, Splenic Fluid
Erythromycin MIC	Sensitive	Blood, Splenic Fluid
Linezolid MIC=4	Sensitive	Blood
Linezolid MIC=2	Sensitive	Splenic Fluid
Penicillin MIC=2	Sensitive	Blood
Penicillin MIC=1	Sensitive	Splenic Fluid
Vancomycin MIC >16	Resistant	Blood, Splenic Fluid

Based on the patient’s previous clinical improvement with IV Piperacillin/Tazobactam and our literature search on *Lactobacillus* susceptibilities, the patient’s course of treatment was changed back to IV Piperacillin/Tazobactam [4]. One dose of IV tobramycin 400mg in sodium chloride was also administered to complete ESBL(+) UTI treatment. The patient then began to improve again and was ultimately able to be discharged back to his nursing home on piperacillin/tazobactam with a percutaneous drainage catheter placed into the perisplenic fluid. Recommendations followed, including continuing both drainage and antibiotics for 1-2 additional months. As interval imaging revealed a significant reduction but not the resolution of the perisplenic fluid, antibiotics, and drainage were completed two months after discharge from the hospital. His leukocytosis improved dramatically. The patient additionally received a thoracentesis for his noted pleural effusion. 

## Discussion

*Lactobacilli, *in general, continue to be recognized as an important bacterial group in multiple fields. The roles that these bacteria play in food fermentation, gut flora, and agriculture are well-studied and recognized [1,4]. However, there is continued recognition that certain species of lactobacilli have a more strained relationship with human hosts. A 2020 review of *L. reuteri* recognizes that this strain instigated both immunological and health benefits, while other species have been shown to colonize internal tissues, including the spleen, much like our patient [5]. This case report demonstrates a novel colonization by *L. rhamnosus* of a splenic abscess and resultant hematoma in a patient with concomitant bacteremia and an ESBL+ UTI. 

*L. rhamnosus*, in particular, strain GG, is generally recognized for its ability to positively impact the microbiome and function of the intestinal tract as it increases IgA secretory cells into the mucosa and antigen transport to lymphoid tissue, promoting the overall health of the intestinal tract [4-6]. However, it is increasingly recognized that *L. rhamnosus,* along with other species of lactobacilli, are pathobionts and may opportunistically infect patients with immunosuppression. This patient was at increased risk of infection with this pathobiont as it was noted that he had many risk factors, including poorly controlled type 2 diabetes, concurrent *E. coli *ESBL+ infection, and renal failure. 

While this case report does have many strengths, including describing a rare splenic abscess and hematoma and subsequent bacteremia with a bacteria more often recognized as a probiotic that is found in cheese, yogurt, and normal gut flora, there are limitations as well. Follow-up with the patient was difficult, as was identifying a source of possible infection, given that the patient himself was a poor historian and resided in a communal nursing home facility. Additionally, further scanning, including a transesophageal echocardiogram, was considered in this patient given the history of ICD placement as a potential seeding source with the recognition that lactobacilli are capable of forming biofilms; however, given the patient's frailty and the risks and benefits of the procedure, this was ultimately abandoned [7]. 

Treatment with IV piperacillin/tazobactam is published to enhance the currently sparse literature on treatment modalities for infections with *Lactobacillus*. Interestingly, meropenem did not confer improvement in the patient and emphasized the idea that susceptibility in lactobacilli is species-dependent [8]. As a result, obtaining blood cultures and determining susceptibility is critical in these infections. Ultimately, the treatment of this large splenic fluid collection and bacteremia was based on our review of the literature and tested susceptibilities in conjunction with the patient's clinical outcome. In this patient’s case, the infection appears to have been an opportunistic pathobiont currently colonizing the patient given his history of diabetes and the fact that he was not on probiotics to our knowledge based on a review of nursing home medication and the patient's statements [9,10]. The use of IV piperacillin/tazobactam as well as drainage of the abscess were necessary for the patient's overall improvement, and repeat imaging four weeks after discharge showed a significant decrease in abscess size. 

## Conclusions

The rarity of lactobacilli-based bacteremia and abscesses, as well as the scarcity of literature regarding the treatment of lactobacilli-based illnesses, prompted an interdisciplinary approach to treating this patient, with pharmacy, infectious disease, interventional radiology, and internal medicine participating in the care. Lactobacilli continue to be a remarkably diverse and evolving group of bacteria, with certain clades and species having an increased risk of virulence in certain populations of patients. This case has shown that native *Lactobacillus rhamnosus *has the ability to infect and colonize splenic hematomas, leading to a systemic complication of bacteremia. We further presented that obtaining susceptibilities as well as draining infected sites were necessary for the clinical improvement of the patient. Further research into the management and treatment of lactobacilli-based illnesses, particularly in high-risk groups such as those with diabetes, will continue to be a necessity.
